# Relationship Between Heart Rate and Perceived Stress in Intensive Care Unit Residents: Exploratory Analysis Using Fitbit Data

**DOI:** 10.2196/60759

**Published:** 2024-11-27

**Authors:** Ruijing Wang, Olya Rezaeian, Onur Asan, Linghan Zhang, Ting Liao

**Affiliations:** 1 Department of Systems and Enterprises Stevens Institute of Technology Hoboken, NJ United States; 2 Department of Industrial Engineering and Innovation Sciences Eindhoven University of Technology Eindhoven Netherlands

**Keywords:** stress, perceived stress, heart rate, Fitbit, wearable, provider, occupational health, resident, trainee, physician, health care worker, intensive care unit, secondary data analysis, mental health, self-reported

## Abstract

**Background:**

Intensive care unit (ICU) residents are exposed to high stress levels due to the intense nature of their work, which can impact their mental health and job performance. Heart rate measured through wearable devices has the potential to provide insights into residents’ self-reported stress and aid in developing targeted interventions.

**Objective:**

This exploratory study aims to analyze continuous heart rate data and self-reported stress levels and stressors in ICU residents to examine correlations between physiological responses, stress levels, and daily stressors reported.

**Methods:**

A secondary data analysis was conducted on heart rate measurements and stress assessments collected from 57 ICU residents over a 3-week period using Fitbit Charge 3 devices. These devices captured continuous physiological data alongside daily surveys that assessed stress levels and identified stressors. The study used Spearman rank correlation, point-biserial correlation analysis, 2-tailed paired *t* tests, and mixed-effect models to analyze the relationship between heart rate features and stress indicators.

**Results:**

The findings reveal complex interactions between stress levels and heart rate patterns. The correlation analysis between stress levels and median heart rate values across different percentile ranges showed that lower percentile heart rates (bottom 5%, 10%, 25%, and 50%) had modest correlations with stress, whereas higher percentiles (top 50%, 25%, 10%, and 5%) did not correlate significantly (all *P*>.05). The 2-tailed paired *t* test indicated significant differences in stress levels reported in midday versus end-of-day surveys (*P*<.001), although these changes in stress levels were not consistently reflected in heart rate patterns. Additionally, we explored and found that stressors related to “other health” issues had the highest positive correlation with stress level changes from midday to end-of-day surveys. However, the weak effect of these stressors on peak heart rate suggests that their impact on physiological measures like heart rate is not yet clear. According to our mixed-effects model, stress levels significantly influenced heart rate variations when hierarchical data were taken into account (*P*=.03), meaning that as the stress level increased, there was a significant increase in mean heart rate.

**Conclusions:**

This study highlights the complexity of using heart rate as an indicator of stress, particularly in high-stress environments like the ICU. Our findings suggest that while heart rate is found to correlate with self-reported stress in the mixed-effect model, its impact is modest, and it should be combined with other physiological and psychological measures to obtain a more accurate and comprehensive assessment of residents’ stress levels.

## Introduction

### Background

The intensive care unit (ICU) is a specialized hospital department that provides intensive and constant treatment and monitoring to patients with severe or life-threatening illnesses and injuries. This necessitates a versatile ICU staff team of competent experts skilled in different treatments and advanced medical techniques and their seamless collaborations [1]. Moreover, these staff members face unpredictable, challenging, and high-demanding tasks in their everyday work while frequently encountering traumatic and ethical issues; making critical end-of-life decisions; visualizing open wounds, massive bleeding, and deaths; dealing with combative family and patients; and providing postmortem care [2]. As a result, it is widely recognized that more ICU staff feel stressed, overwhelmed, and burned out compared to hospital physicians, eventually jeopardizing their job satisfaction, well-being, and quality of care [3]. According to a study by Kumar et al [4], the prevalence of stress among ICU nurses and doctors is 52.43%.

Among ICU staff, residents are an especially high-risk group for stress and burnout. Residents are also called trainee physicians who have finished medical school and taken postgraduate training and clinical roles at hospitals. On top of their highly intensive training and demanding tasks at the ICU, these trainee physicians also generally struggle with more uncertainties and stressors that arise from both work and personal lives [5]. A line of related research from the Association of Program Directors in Internal Medicine in the United States categorized residents’ stressors into 3 classes: situational stressors like excessive workload; personal stressors like financial issues; and professional stressors like examination and career planning [6]. Furthermore, residents are generally less experienced in stress coping as no formal training is provided, and they lack supportive resources [7]. Several studies show that residents tolerate higher and more severe stress than senior physicians [8,9]. This not only affects the residents’ performance and well-being but also leads to more medical errors.

Due to the severity of this issue, researchers are calling for appropriate interventions to address residents’ stress [10-12]. Such appropriate interventions are based on the premise of precise identification of stress sources, on-time intervention, and personalized intervention methods [10]. Despite extensive research on residents’ stress, stressors, and burnout, almost all existing research focuses on either qualitative research methods or questionnaires that only enable the identification of resident stress based on their daily or even 1-time self-reflection and reports. On the one hand, such sparse, subjective data collection cannot be used to track the real-time stress of the residents and thus fails to support timely intervention; on the other hand, it can be mistaken as it depends on the residents’ memory and hence causes inadequate intervention.

One ecologically valid solution for real-time stress tracking is via wearables. With advances in sensors, the Internet of Things, sensing technology, and artificial intelligence, wearables like wristbands (eg, Fitbit), smartwatches (Apple and Samsung smartwatches), smart rings (Oura rings), and smart belts (Polar belt) are capable of tracking physiological signals like heart rate (HR), HR variability (HRV), electrodermal activity, body temperature, and body movements (via accelerometers) constantly, and based on which, computing and reporting human status in real-time. In recent years, these devices have been widely used by individuals keen to understand and promote their physical (eg, sleep tracking, step counting) and mental health (eg, stress tracking). Indeed, physiological signals reflect the status of the autonomic nervous system (ANS), which is related to the involuntary or unconscious processes of the human body, such as heart beating. Specifically, the ANS can be further divided into sympathetic nervous system (SNS) and parasympathetic nervous system (PSNS). The former is responsible for our “fight-or-flight” instincts when the human brain perceives danger and thus triggers acute stress responses like increased HR and heart contraction forces; the latter helps to conserve and restore the stressed status and main “rest-and-digest” of the human body. The SNS and PSNS work synergetically, creating balancing acts and smooth transitions when humans deal with different environmental conditions. Therefore, tracking the ANS system, or features of the ANS system like HR (or other physiological signals), enables understanding of the dominant (SNS or PSNS) system and implies human status like stress.

Indeed, existing research shows that physiological signals like HRV are highly related to stress [13]. For instance, Taelman et al [14] compared the HR and HRV of 28 participants at rest or with mental stressors and concluded that HR and HRV may have the potential to measure stress levels. Moreover, Schubert et al [15] found that chronic and short-term stress can affect different HR and HRV metrics. However, although promising, applying physiological signal- or wearable-based stress computing, or in general, affective computing, is extremely challenging since most existing studies on affective computing conducted their experiments in controlled laboratory settings where experimental stimuli are relatively simple, monotonous, nonrealistic situations (eg, “imagine you are going to have your final exam in 10 minutes”), and the participants are normally required to be stationary. Such settings aim to avoid complex physiological signal changes and noises introduced by the surroundings or participants (eg, movements, skin tone). As a result, the conclusions drawn from such experiments can be related to those in naturalistic settings or in the wild but less convincing. Physiological signal-based stress computing in naturalistic settings is still an open and challenging issue, especially given that ICU residents work in complex environments. Nevertheless, this paper evaluates long-term HR data collected from ICU residents in a 3-week study period. Such an extensive dataset and continuous data support the chances of improved noise reduction and the possibilities of stress-caused physiological signal change identification.

### Objective

This exploratory study analyzed long-term HR data collected by wearable devices and survey responses from ICU residents in a clinical setting. It aimed to investigate the correlation between HR and self-reported stress levels and assess the influence of daily stressors, contributing to a deeper understanding of how stress varies in clinical settings. This understanding could inform future strategies to support health care professionals, potentially enhancing decision-making and patient care.

## Methods

### Study Design and Dataset

This study conducted a secondary data analysis of the dataset, which was collected from 57 residents enrolled in the ICU rotation at the Los Angeles County and University of Southern California Medical Center between November 2019 and March 2020 [16]. Participating residents were provided with a Fitbit Charge 3, and they wore the device continuously over the 3-week study period. The device recorded physiological data, including HR, step count, sleep duration, and sleep quality.

Two daily surveys were administered during the study for stress assessments. The midday survey was issued at 12:15 PM each day, and participants retrospectively rated their stress level for the morning hours on a 7-point Likert scale ranging from 1=not at all stressed to 7=extremely stressed. The end-of-day survey was deployed at 6:15 PM, which extended the assessment to afternoon stress levels and included questions on perceived daily stressors, work behaviors, and sleep quality. The surveys also allowed participants to specify stressors encountered and categorize them as personal (eg, financial issues, health problems) and professional (eg, interpersonal conflicts, discrimination) domains. To provide an overview of the sample characteristics, the descriptive statistics of the key demographic variables of the study participants are summarized in Table 1.

**Table 1:** Descriptive statistics of ICU^a^ residents participating in the study (N=57).

Variables			Value
**Age (years), mean (SD; range)**			29.4 (2.3; 25-34)
**Years of residency, mean (SD; range)**			2.1 (0.9; 1-4)
**Sex, n (%)**			
	Male	34 (60)	
	Female	23 (40)	

^a^ICU: intensive care unit.

### Ethical Considerations

The original data collection for the study was conducted under the institutional review board approval obtained by the authors of the TILES-2019 dataset (HS-19-00606). All participants in the original study provided informed consent. The data used in this study were fully anonymized before analysis, ensuring that no personal identifiers were included. Data confidentiality was maintained throughout the research process. Participants in the original study were compensated according to the guidelines of the primary research protocol. Focal participants were compensated with a gift card and were allowed to keep the Fitbit. No additional compensation was provided for this secondary analysis.

### Data Processing

**Figure 1:**
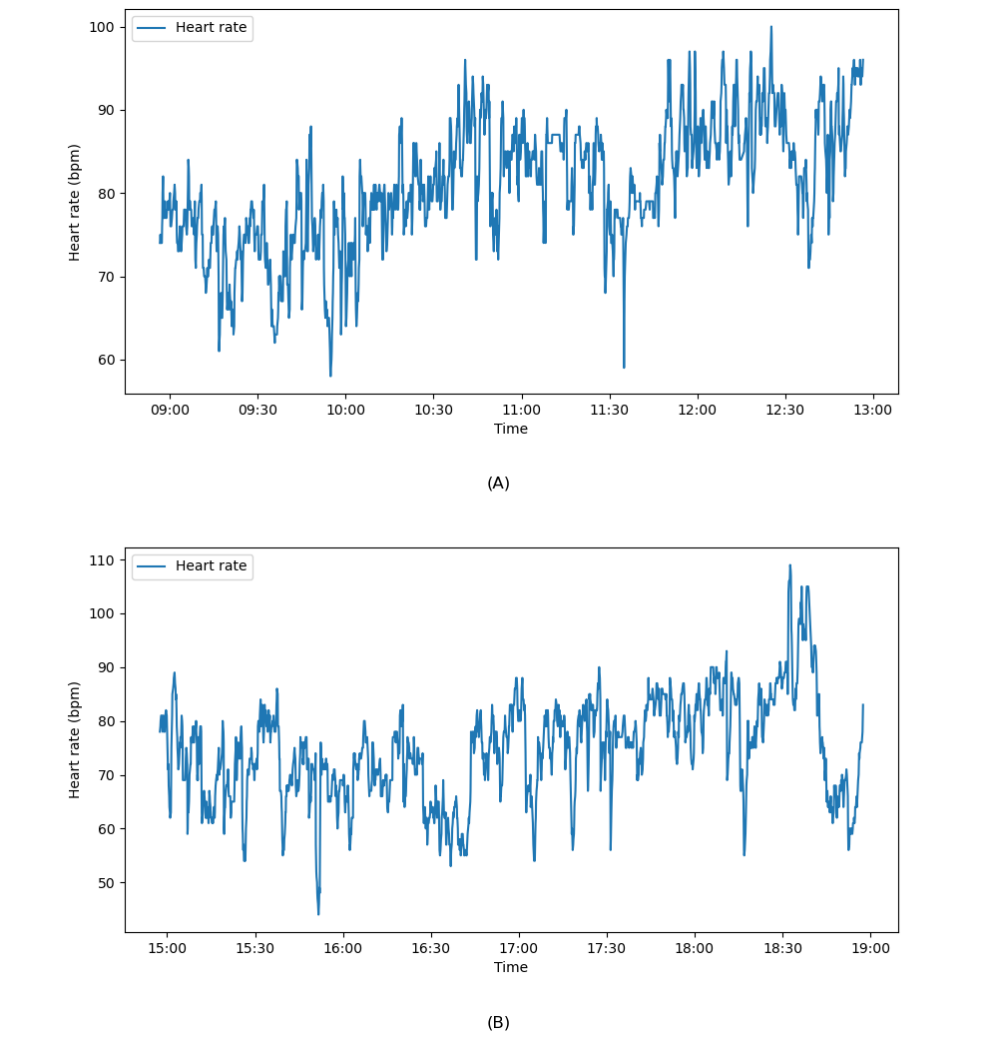
Raw heart rate data before midday and end-of-day surveys for 1 participant on January 14, 2024: (A) before midday survey (stress level 3.0) and (B) before end-of-day survey (stress level 2.0). bpm: beats per minute.

HR data were reported at nonuniform intervals anywhere between approximately 5 seconds and 15 minutes, depending on the participants’ physical activity [16]. For data cleanup and preprocessing, all empty files and duplicated columns that occurred in some files were removed. We matched the HR dataset with the corresponding reported stressor and stress level dataset for each participant and then excluded participants who were missing either the HR dataset or the reported stress dataset. After preprocessing, 50 complete survey responses were obtained with matching HR files. To facilitate comparative analyses between participants, we normalized HR data using a minimum-maximum normalization technique [17], which adjusts HR values to a standardized range of values without distorting differences in value ranges. After normalization, we aligned the timestamps of the HR data and survey responses to synchronize physiological data with stress assessments. Finally, due to the different time intervals of the midday and end-of-day surveys (4-hour vs 6-hour), we extracted HR data for a 4-hour window preceding each survey time to maintain a consistent data structure. After these steps, the dataset contains each participant’s raw HR data before the midday and end-of-day surveys with the corresponding stress level, for example, shown in Figure 1. The raw HR data exhibit noise, necessitating the identification of key features for extracting essential information for analysis.

### Feature Selection of HR Data

To extract essential information from the HR data, fundamental statistical terms were first calculated at the aggregated level. The key metrics for assessing the cardiac status of ICU residents include mean HR, minimum HR, median normalized HR, HR SD, and the ratio of HR above 100 bpm [18-20].

The median HR, a common measure of central tendency, has been widely used in various studies [21,22]. Moreover, previous research shows inconclusive results in the complicated correlation between objective and continuous (4-hour window, multiple samples per minute) physiological measurements like HR and subjective and discrete self-reported mental health data [23]. Such discrepancies can reside in the biased or falsely self-reported stress levels affected by cognitive processes, social desirability, and survey conditions [24]. Alternatively, they can also be affected by various random noises in the physiological signals caused by environmental or context factors like temperature [25], light [26], individuals’ circadian rhythms [27], human activities [28], or drinking coffee [29]; extraordinary experiences like winning a lottery; and underlying mechanism or inferior quality of the sensors [30,31]. Moreover, the HR data reflect the continuous changes over 4 hours. In contrast, the survey data were only collected twice daily in this study, which can be biased by individuals’ memory recall [14]. Therefore, to mitigate the influences of various biases, errors, and noises in the data and to gain insights into the complex correlation between perceived stress and HR, we decided to use the median of different percentiles of the HR data. Specifically, in addition to the median of the entire 4-hour window, we experimented with specified percentile ranges for each participant, specifically the top and bottom 5%, 10%, 25%, and 50%, which were calculated to characterize the central tendency of HR distribution. The median values potentially also provide insights into the distribution and variability of HR responses across participants.

According to the existing literature on physiological markers of stress [32], an advanced measurement, peak HR, was generated to represent the highest HR during the 4-hour window preceding each survey response. It captures moments of acute physiological arousal that may be associated with stressful experiences.

### Statistical Analysis

This study used a list of statistical analyses in 3 stages to first explore the relationship between long-term HR and stress reported by ICU residents and also examine the potential factors for stress in the clinic setting. In stage 1, we conducted the correlation analysis to explore the relationship between key cardiac status metrics and self-reported stress levels. In addition, we analyzed the correlations between the median HR across different percentile ranges and stress levels, aiming to determine which percentile ranges showed more pronounced correlations with stress. Following this inspiration, we also investigated whether the HR pattern differs in the midday and end-of-day periods by extracting HR readings from the 4-hour periods before each survey’s timestamp. This approach allowed us to analyze how immediate physiological conditions, captured through HR, correlated with the stress levels reported by ICU residents at different times of the day.

To understand the causes of stress in stage 2, we performed 2-tailed paired *t* tests on stress levels reported in the midday and end-of-day surveys for each day. In the next step, we examined the influence of various daily stressors on stress levels and physiological responses, particularly peak HR. Through this, we aimed to understand the influence of day-to-day challenges on the residents’ stress experiences.

Furthermore, in stage 3, we applied mixed-effect models to accommodate the nested structure of the data and account for the correlation of repeated measurements from the same individual, which usually exists in longitudinal data. The model incorporates median HR, stress level, and survey type (midday or end-of-day).

The mean HR was entered as the dependent variable in the mixed-effects model, with stress level and survey type as the fixed effects. We added random intercepts for each participant in the model, trying to capture individual variability in HR. Those random intercepts acknowledge and account for the inherent differences in physiological baseline found from person to person. Additionally, we considered the random slopes for the survey type, exploring whether the timing of the survey influenced HR across participants, thus allowing for personalized response patterns.

Each stage of our analysis deepens our understanding of the complex relationship between the physiological characteristics of stress and the subjective experience of ICU residents. More sophisticated statistical techniques are used, culminating in mixed-effects models, allowing us to distinguish between fixed and random effects that contribute to HR, thus providing a comprehensive insight into the phenomenon under study.

## Results

### Stage 1: Correlation Analysis Between HR Metrics and Stress Levels

We used Spearman rank correlation analysis to examine the linear relationship between key cardiac status metrics and self-reported stress levels among ICU residents, with correlation coefficient values ranging from –1 to 1 [33].

A significant positive correlation (0.14; *P*<.001) was found between mean HR and stress levels, shown in Table 2. The minimum value and SD of HR are also found to be significantly correlated with stress levels (both *P*<.001). However, the correlation coefficients between these metrics and stress levels are small, and the association may not be particularly relevant. This indicates that HR, as measured through these fundamental statistical terms, may not serve as a strong stand-alone indicator of stress within the context of ICU residents.

**Table 2:** Correlation coefficients between heart rate metrics and stress levels.

Heart rate metrics	Coefficient	*P* value
Mean heart rate	0.1401	<.001
Minimum heart rate	0.1068	<.001
Normalized mean heart rate	0.0609	.02
Normalized median heart rate	0.0639	.11
Ratio of heart rate above 100 (bpm^a^)	0.0289	.28
Maximum heart rate	0.0237	.37
SD of heart rate	–0.1036	<.001

^a^bpm: beats per minute.

Beyond the fundamental statistical terms, we explored the correlation between stress levels and median HR values of top and bottom 5%, 10%, 25%, and 50% percentiles to identify whether HR segments are associated with self-reported stress levels among ICU residents. We used Spearman rank correlation analysis for stress levels on an ordinal scale. There is no significant correlation between the median values and stress levels for the upper percentiles of values, as shown in Table 3 (all *P*>.05).

**Table 3:** Correlation between upper percentiles median heart rate and stress levels.

Heart rate percentiles	Correlation coefficient	*P* value
Top 5% median heart rate	0.0227	.39
Top 10% median heart rate	0.0248	.35
Top 25% median heart rate	0.0239	.37
Top 50% median heart rate	0.0297	.26

For the lower percentiles, the correlations are tested to be significant (all *P*<.05), as shown in Table 4, whereas the correlation coefficients, ranging from 0.0967 to 0.1083, are relatively small. The low coefficients indicate a weak linear relationship between the lower percentiles of median HR and stress levels. These weak but significant correlations may be due to the large sample size of testing or other important but unrecognized confounding factors of median HR. Although the associations between the lower percentiles of median HR and stress levels are tested to be significant and have greater magnitude compared to the upper segments, the results suggest that stress levels may not be reflected by median HR levels.

**Table 4:** Correlation between lower percentiles median heart rate and stress levels.

Heart rate percentiles	Correlation coefficient	*P* value
Bottom 5% median heart rate	0.1066	<.001
Bottom 10% median heart rate	0.1083	<.001
Bottom 25% median heart rate	0.1046	<.001
Bottom 50% median heart rate	0.0967	.002

**Figure 2:**
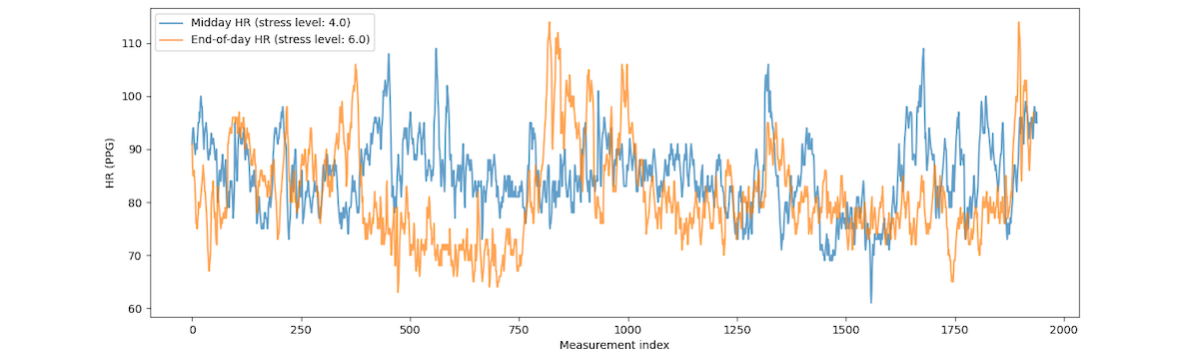
Comparison of HR patterns between midday and end-of-day surveys for 1 participant. HR: heart rate; PPG: photoplethysmography.

Similar to initial expectations, higher reported stress levels did not generally correspond to higher metric values during the measuring window. This can be further reflected by a representative case shown in Figure 2, which illustrates the HR of 2 entire windows of 1 participant for 1 day. The patterns can hardly be distinguished given the significantly different stress levels (6 vs 4). Regarding the period right before the survey (1500th-1700th samples), the HRs of higher stress (blue) are lower than those of lower stress (orange line). This observation suggests that the relationship between immediate physiological responses (measured by raw HR) and subjective stress experience is more complex than direct causation.

### Stage 2: Analysis of Stress Level Differences and Relevant Factors

In the second stage of analysis, we explored the differences in stress levels reported in midday and end-of-day surveys among ICU residents and compared corresponding HR patterns. We performed a 2-tailed paired *t* test on a significant sample of 669 instances of completion of both surveys [34]. Specifically, the *t* test was conducted to compare the absolute value of the difference in stress levels reported between the 2 survey times against 0. This approach allowed us to assess whether the magnitude of stress level changes was significant, regardless of the direction of change. The 2-tailed *t* test analysis yields a *t* statistic of 21.02 and a *P* value of <.001. The result indicates that the stress differences between midday and end-of-day surveys observed in the dataset are statistically significant. However, we did not observe that 1 survey time consistently resulted in higher stress levels than the other. The range of variability in stress experiences, with both positive and negative differences between the midday and end-of-day surveys observed across the study population, indicates no consistent trend in stress levels at either time of day.

Despite significantly different stress levels between midday and end-of-day surveys, the HRs measured did not demonstrate significant differences according to the previous analysis (Figure 2). In addition to the survey type or time, we examined the specific stressors reported by ICU residents and their influence on changes in their stress levels and peak HRs. To report daily stressors, participants indicated whether and how frequently they had experienced stress that day from any of the sources in Table 5. All reported stressors are nonprofessional as they pertain to personal and family-related issues rather than professional or clinical activities.

**Table 5:** Meaning and frequency of daily stressors reported by participants.

Daily stressor	Meaning	Frequency, n
Partner	Tension or arguments with spouse or partner	10
Family	Tension or arguments with family members	5
Breakdown	An item breaking	20
Money	Not having enough money to pay bills, loans, or something else that is needed	15
Self-care	Finding time for self-care	60
Health	Own health problems	30
Other health	Someone else’s health problems	40
Household	Doing or needing to do household tasks	25
Child	Caring or arranging care for the child	5
Discrimination	Experiencing discrimination	2
None	None	280

Self-care is the most prevalent stressor, indicating that ICU physicians are consciously aware of the challenges. Yet, participants selected “none” 280 times, even though they worked under substantial stress.

To investigate the influence of the reported stressors, we computed the point-biserial correlation coefficient between each stressor and the magnitude of change in stress levels between midday and the end-of-day surveys. In the analysis, the stressors were encoded as binary states indicating the presence or absence of each stressor. The point-biserial correlation is used to measure the relationship between a continuous variable and a dichotomous variable. According to the study design, stressors were reported only in the end-of-day surveys, but stress levels were assessed at midday and at the end of the day. Participants presumably recalled stressors that happened close to the end of day in their short-term memory, and the change in stress levels is likely to capture the cumulative impact of daily stressors. Thus, we focused on analyzing changes in stress levels rather than comparing raw stress values. The correlation analysis shows that “other health,” which involves health issues of others other than participating ICU residents, emerged as the stressor with the highest positive correlation (0.1694) with stress level, shown in Table 6. On the other hand, “none” showed a statistically significant negative correlation (–0.1085; *P*=.005) with stress level changes, indicating that participants reported less change in stress on days without any specified stressor. Yet, due to the small coefficient values, the correlations between the stressors and stress levels are generally weak, and the significance may be due to other unknown confounding factors.

**Table 6:** Correlations between daily stressors and stress level changes.

Daily stressor	Coefficient	*P* value
Partner	–0.0756	.0504
Family	0.0996	.009
Breakdown	–0.0741	.055
Money	0.0062	.87
Self-care	–0.033	.39
Health	0.0098	.80
Other health	0.1694	<.001
Household	–0.0386	.32
Child	N/A^a^	N/A
Discrimination	0.0391	.31
None	–0.1085	.005

^a^N/A: not available.

Moreover, when we assessed the impact of the stressors on changes in stress levels through regression analysis, the results showed that the model’s explanatory power was relatively low, with a mean squared error of 0.638 and an *R* squared value of 0.012. It can be inferred that other unexamined factors, in addition to the predetermined stressors in the survey, may explain variations in stress levels among ICU residents.

Finally, inspired by the literature [35], we examined the relationship between reported stressors and peak HR using linear regression. The regression results show that the stressors “partner” and “breakdown” have significant effects on the peak HR (both *P*<.001), whereas the coefficient is close to 0 (Table 7). This indicates no meaningful changes in peak HR associated with partner-related stress. Other stressors, such as “family” (*P*=.03), “other health” (*P*<.001), and “household” (*P*=.003), also showed statistically significant effects and more substantial influence on the peak HR.

**Table 7:** Summary of linear regression results for stressors and peak heart rate.

Variable	Coefficient	*t* value	*P* value >|*t*|
Partner	1.03×10^16^	3.595	<.001
Family	0.2134	2.175	.03
Breakdown	1.203×10^–16^	3.757	<.001
Money	1.016×10^–16^	2.846	.005
Self-care	–0.0115	–0.397	.69
Health	–0.1119	–1.980	.049
Other health	–0.0982	–3.356	<.001
Household	0.2477	2.984	.003
Child	N/A^a^	N/A	N/A
Discrimination	–0.0749	–0.765	.44
None	–0.0435	–1.965	.051

^a^N/A: not available.

The model has several limitations. The *R* squared value is 0.110, suggesting that the model does not explain much of the variation in the data. However, the statistical significance of the model was confirmed (*F*_11,491_=5.52, *P*<.001) for the collective relationship between the identified stressors and peak HR. The model also produces a high condition number (5.68×10^33^), indicating potential problems with the multicollinearity of the stressors. Although providing insights into the causes of daily stress, the model needs further enhancement to disentangle these relationships.

### Stage 3: Mixed-Effects Model of Stress, HR, and Relevant Factors

In stage 3, we implemented the mixed-effects model to investigate the influence of stress level and survey type on mean HR while accounting for the nested structure of the data with the repeated measurements of the study. The model was applied to data consisting of 50 unique participants with 1419 observations. Each participant was considered a separate group. The observation size ranged from 1 to 43 in each group, while the average size for observations recorded was 28.4. In our analysis, the intercept is 80.77, shown in Tables 8 and 9. This means the HR is approximately 80 beats per minute at noon for individuals under low stress. This number served as a starting point for understanding how HR changed under different conditions. The high level of certainty (*P*<.001) in this result means that it is unlikely this is a random finding.

**Table 8:** Results of the linear mixed model on the influence of stress level and survey type on mean heart rate (part 1).

Model	Mixed linear model	Dependent variable	Mean heart rate
Number of observations	1419	Method	REML^a^
Number of groups	50	Scale	61.99
Minimum group size	1	Log-likelihood	–5023.08
Maximum group size	43	Converged	yes
Mean group size	28.4	—^b^	—

^a^REML: restricted maximum likelihood.

^b^Not applicable.

**Table 9:** Results of the linear mixed model on the influence of stress level and survey type on mean heart rate (part 2).

	Coefficient	SE	*z*	*P* value >|*z*|	[0.025	0.975]
Intercept	80.75	1.2	67.09	<.001	78.41	83.13
Suvey_Type [end-of-day]	–0.64	0.47	–1.36	.17	–1.57	0.28
Stress_Level	0.33	0.15	1.28	.03	0.03	0.63
Group heart rate variance	56.31	1.58	—^a^	—	—	—
Group heart rate and Survey_Type [end-of-day] covariance	-0.35	0.47	—	—	—	—
Survey_Type [end-of-day] variance	1.67	0.26	—	—	—	—

^a^Not applicable.

**Figure 3:**
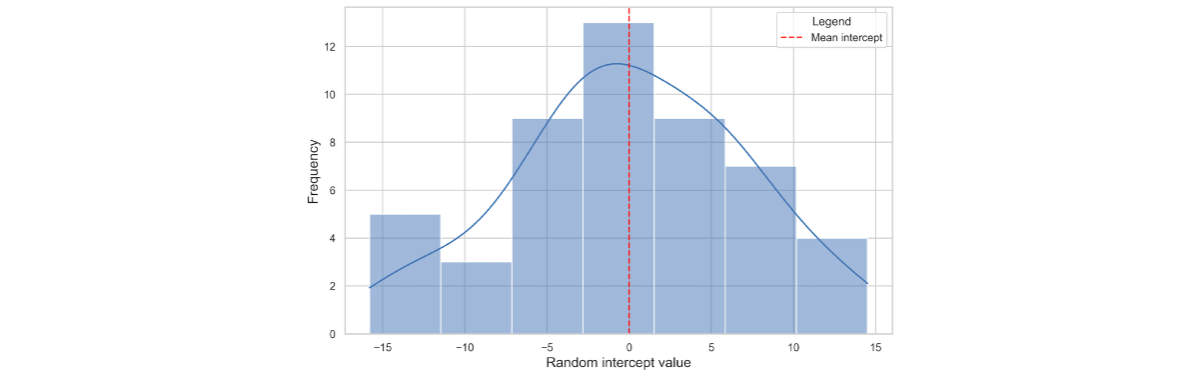
Distribution of random intercepts in the mixed-effects model for heart rate.

The individual differences in physiological responses are illustrated as a histogram of random intercepts from the mixed-effects model shown in Figure 3. This histogram shows a normal distribution centered around a mean close to 0, highlighting interindividual variability in baseline HR among participants. The distribution spans approximately from –15 to +15, emphasizing differences in baseline physiological states across individuals. Such variability underscores the importance of accounting for individual differences when analyzing physiological responses, as it confirms that baseline HR levels can influence the observed effects of stress levels and time of day on HR measurements across a diverse participant pool.

**Figure 4:**
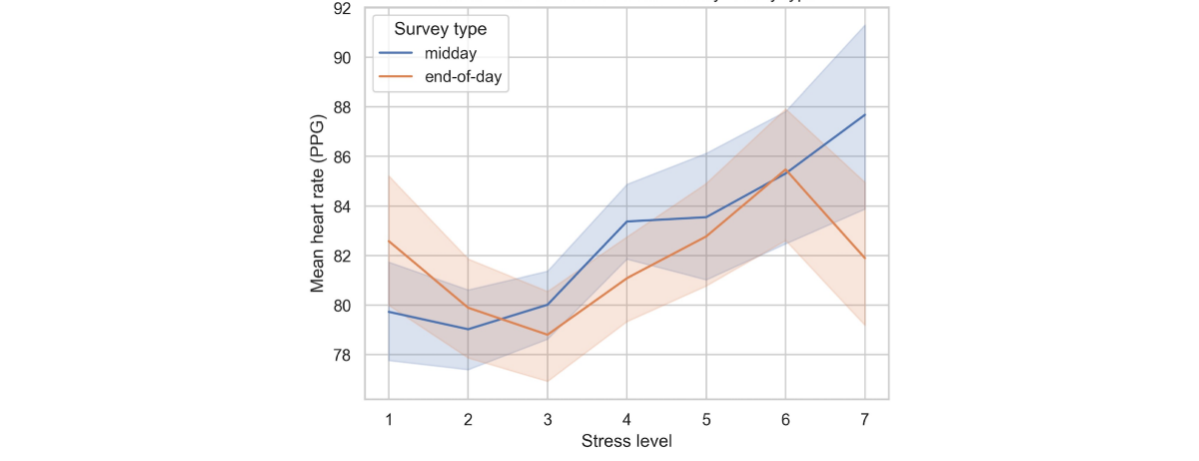
Impact of stress level on heart rate by survey type. PPG: photoplethysmography.

The impact of survey type on HR revealed distinct patterns throughout the day. Compared to that at midday, HRs at the end of the day are generally lower, with a decrease of 0.644 beats per minute (*P*=.17); however, this difference was not statistically significant. As shown in Figure 4, mean HRs demonstrated an approximately linear trend for the stress levels in the midday surveys; mean HRs that corresponded to the end-of-day surveys fluctuated, particularly for low stress and high stress. Individual differences in HR responses to survey type are evident in variances of 1.677 (SE 0.265) for the end-of-day surveys. It seems that even though time of day may affect responses to stress, it varies significantly among individuals, with some showing heightened sensitivity to survey type.

The result in Table 9 demonstrates a significant relationship between participants’ stress levels and HRs. The model estimated that for each unit increase in stress level, there is a corresponding increase in mean HR of 0.334 beats per minute (*P*=.03). The strong correlation between perceived stress level and HR is statistically significant, confirming that higher stress levels are consistently associated with higher HRs.

The random effects analysis within our mixed-effects model reveals substantial variability in HR responses among participants, as indicated by the group variance of 56.313. As a result of all this interindividual variability, baseline HRs differ significantly across participants (*P*<.001), highlighting the need to monitor and manage HRs individually. In addition, the variance component for the end-of-day survey type is 1.67, suggesting that HRs vary at different times of day and in different ways. Moreover, the covariance between individuals’ baseline HRs and how those rates change at the end of the day shows a negative value of –0.349, indicating an inverse relationship. Those with lower HRs at the start might have more substantial increases by the end of the day, while those starting with higher rates might not experience as much change.

## Discussion

### Principal Findings

This secondary analysis study has produced several findings with implications for understanding stress in clinical environments. Our initial correlation analysis suggested a modest correlation between average HR and stress levels. This is consistent with previous research indicating that HR may be a more complex indicator of stress than previously thought [36]. The weak correlations across key metrics highlight the potential limitations of using HR alone as an indicator of stress, echoing the findings of Matsumoto et al [37] on the need to combine biometric data with other subjective measures for a comprehensive stress assessment. In addition, the metrics extracted from the entire 4-hour window provide aggregated information, which may not explain the instantaneous stress reported in surveys. Further analysis is necessary to understand when and how much physiological history needs to be collected for stress analysis.

Beyond fundamental metrics, the median HR in lower percentiles (5%, 10%, 25%, and 50%) showed low-to-moderate positive correlations with stress levels. The result shows that self-reported stress can be captured by the lower end of the HR distribution. However, median HRs of the upper percentiles were very weakly correlated with stress levels. This highlights the complexity of the stress response, as these upper percentile HR values were not significantly consistent with residents’ self-reported stress. As suggested by Clarke et al [38], this may be due to a variety of factors, including individual differences in stress physiology or the presence of nonstress-related physiological factors. Psychological factors such as reporting biases or professional norms that encourage underreporting stress might contribute to these findings. External factors like medication or caffeine intake and the specifics of the measurement methodology could also play significant roles [39,40]. Relying solely on traditional HR metrics may not sufficiently capture the multifaceted nature of stress responses in health care settings. Therefore, future research should consider these variables to better understand stress physiology in clinical environments.

Further examination of significant differences in stress levels reported at different times of the day, as revealed by the 2-tailed *t* test analysis, indicates the dynamic nature of stress in health care professionals, confirming the study of Vamvakas et al [41]. Interestingly, higher stress levels did not consistently correlate with higher median and peak HRs. This result suggests the need for further investigation into additional metrics such as HRV, cortisol levels, and skin conductance, which may also significantly influence these correlations and provide a more comprehensive understanding of physiological responses to stress [42,43]. The lack of a consistent correlation between higher stress levels and higher HRs challenges the direct relationship assumed in early stress models [36]. This finding aligns with recent work by Sommerfeldt et al [44], which shows individual differences in physiological stress responses and suggests that future studies should focus on establishing personalized profiles that account for such variability.

The implementation of our mixed-effects model provided further insight into these dynamics by accounting for the nested structure of the data, a crucial aspect given the repeated measures across subjects [45-47]. As a result of this modeling approach, we found that, when the hierarchical nature of the data was considered, stress levels were significantly correlated with HR variations, contradicting initial correlation analyses. According to the mixed-effect model, each unit increase in stress level corresponded to a 0.334 beat per minute increase in mean HR (*P*=.03) among participants. The variability in HR responses, as indicated by the group variance of 56.313 and the specific patterns observed in responses to different times of day, further support the notion that individualized approaches are critical in managing health outcomes in high-stress environments like health care.

Specific stressors, particularly those related to the health of others, had more pronounced effects on stress levels, perhaps reflecting the inherently empathetic nature of health care work [38,48]. In contrast, days without specific stressors reported less change in stress, but this does not necessarily imply consistently lower stress levels on such days, challenging the notion of accumulation of stress through identifiable daily stressors alone. This is consistent with the view proposed by previous literature, which argues that stress may also arise from a lack of stressors, as this may indicate insufficient stimulation or lack of engagement [48,49].

These findings collectively suggest a complex interplay between physiological measures and the psychological landscape of stress. This aligns with the idea that stress in clinical settings is a dynamic and complex experience impacted by individual, situational, and emotional factors [50]. The results highlight the importance of considering the broader context of individual experiences and the multidimensionality of stressors in assessing and managing stress among health care professionals. The study sets a foundation for future research further to unravel the intricacies of the HR-stress relationship, incorporating additional variables like sleep quality and workload to enhance stress management strategies within high-pressure clinical settings.

### Limitations

The modest sample size and the focus on ICU residents may limit the generalizability of our results to other settings or populations. Additionally, the study relied on self-reported measures of stress, which are subjective and may be influenced by individual variability in stress perception and reporting accuracy. The use of wearable technology has some constraints. The Fitbit Charge 3 may not capture the full complexity of physiological responses to stress. Also, factors such as device placement, physical activity, and external environmental influences could affect the accuracy of the data collected. Future research incorporating a wider range of physical, physiological, and psychosocial variables may improve the understanding and predictive capability for stress levels in clinical environments. The demographic factors that potentially influence stress levels may be further examined to provide a deeper understanding and guidelines for stress management.

### Conclusions

This study provides valuable insights into the complex relationship between HR and perceived stress levels in ICU residents. We highlight the multifaceted nature of stress responses in high-stress clinical settings by analyzing long-term HR data and self-reported stress levels and stressors. Our findings suggest that while HR indicators can provide some indication of stress, they are not stand-alone indicators and should be combined with comprehensive physiological and psychological measures to obtain a more accurate assessment.

## Abbreviations

ANS: autonomic nervous system.
HR: heart rate
HRV: heart rate variability
ICU: intensive care unit
PNS: parasympathetic nervous system
SNS: sympathetic nervous system

## Data Availability

The data sets analyzed during this study are available in the TILES repository [16].
